# A Case Report of Prolonged Apnea during ECT in a Patient with Suicidal Attempt by Organophosphorus Poison

**Published:** 2012

**Authors:** Sussan Moudi, Ebrahim Alijanpour, Ali-asghar Manouchehri, Hasan Jafarian

**Affiliations:** 1 Assistant professor of psychiatry, Babol University of Medical Sciences.; 2 Assistant professor of Anesthesiologist, Babol University of Medical Sciences.; 3 Forensic medicine and toxicologist, Assistant professor, Babol University of Medical Sciences.; 4 Anesthesiologist, Department of Anesthesiology, Yahya Nejad Hospital, Babol University of Medical Sciences, Babol, Iran.

**Keywords:** Apnea, Electroconvulsive Therapy, Organophosphorus

## Abstract

Organophosphorus pesticides have been used in some cases for suicidal attempts. Such poison can affect plasma cholinesterase activity. The case was a 47-year-old man hospitalized due to suicide attempt with swallowing agricultural poison. The patient, diagnosed with major depressive disorder (MDD), underwent treatment with electroconvulsive therapy (ECT). At the first ECT session, the patient developed apnea for 45 minutes following receiving 20 mg succinylcholine. The patient was intubated; after restoration of respiration depth and rate, the patient was extubated. Collectively, in cases with history of suicide attempts, taking organophosphorus pesticides should be warn for pre-ECT anesthesia.

## Introduction

Since the last few decades, organophosphorus has been used as an insecticide (1). This agent has been used for a long period as pesticide or herbicide in Mazandaran province due to abundant farm fields (2). Organophosphorus toxicity is generally caused by intentional use or accidental exposure to agricultural products or pesticides (3, 4). It is estimated that 3 million people are annually exposed to organophosphorus poisoning around the world and 300 thousands die of this poisoning (5,6).

Organophosphorus poisons are well absorbed through the skin, lungs and digestive system, and bind to acetylcholine esterase in red blood cells as well as nerve terminals leading to inactivation of this enzyme (7). The effect of organophosphorus poisons on nerves is mainly owing to acetylcholine esterase inhibition, which suppresses acetylcholine degeneration in synaptic cleft or nerve-muscle junction and eventuates in persistent neural stimulation and excessive, continuous activation of nicotinic and muscarinic receptors (8). Acetylcholine augmentation in the central nervous system causes anxiety, seizures and apnea (9-12). On the other hand, inhibition of plasma cholinesterase can contribute to increased sensitivity to drugs hydrolyzed by this enzyme, including succinylcholine, mivacurium and chloroprocaine (13). Thus, anesthesia in poison-exposed patients should be taken into consideration.

## Case Report

The case was a 47-year-old man, resident of Babol, illiterate, painter and married who was hospitalized at Yahya Nejad hospital. The patient's problems had begun since the previous year subsequent to stressors (financial problems) accompanied by sadness, distraction, hopelessness, frustration, suicidal thoughts, irritability, sleep deprivation, loss of appetite and decreased performance. The predicament led to referral to psychiatrist, but he did not take the prescribed medicines. He had no history of suicide attempts. Symptoms had been intensified since 9/8/2010, and he had taken the matter up with his family prior to self-poisoning with agricultural toxin on 12/8/2010. The patient did not regret the suicidal attempt, and was taken to the hospital by his family who had been realized by the empty container of the agricultural poison. 

He was then admitted to ICU and was in good general condition during hospitalization till 20/8/2010. Electrolytes as well as blood tests were normal at discharge time

The patient was discharged with drug instructions of ranitidine two times a day and then was hospitalized in Yahya Nejad psychiatric ward. Being diagnosed with major depressive disorder (MDD), he was treated with sertraline plus olanzapine and ECT. In the first session of ECT on 18/8/2010, he developed respiratory apnea for 45 minutes following receiving thiopental 200 mg, and succinylcholine 20 mg. The patient ventilated with ambu and mask and he underwent orotracheal intubation for 45minutes; after restoration of respiration depth and rate, and a normal ABG, the patient was extubated. ABG was ordered an hour later which was normal. In the next ECTs, the case received propofol and atracurium.

## Discussion

Prolonged apnea subsequent to succinylcholine administration has been observed in patients with severely reduced level of acetylcholine esterase or its components (14). Variety of physiological, pharmacological and pathological factors can result in widespread increase or decrease in the enzyme activity. More than 75 percent reduction in the enzyme natural levels will clinically bring prolongation of succinylcholine effect (15). Thus, it seems that our patient, who received 20 mg succinylcholine for ECT six days after organophosphate self-poisoning, developed apnea due to acetylcholinesterase inhibition and alteration in natural process of succinylcholine metabolism under the poison influence. The patient has negative history of pseudocholine esterase deficiency because he received succinylcholine for appendectomy 8years ago. Therefore, this case indicates the importance of pre-ECT screening for organophosphate poisoning, especially when there is a need to apply succinylcholine. Furthermore, regarding that significant proportion of Mazandaran population resides in rural areas with easy access to organophosphate poisons owing to agricultural activities, such issues should be more emphasized by local physicians on agricultural seasons to identify if the patient, supposed to take succinylcholine, has been previously exposed to organophosphorus poisons or not.

Waghmare and colleagues noted that suxamethonium caused prolonged intra-ECT apnea in a patient who had committed suicide with organophosphate (16). Likewise, Sener et al. reported a post-organophosphate-poisoning apnea in a 7-year-old child following subsequent use of succinylcholine. Sener and colleagues mentioned a noteworthy point in their study; the less time between the toxin exposure and succinylcholine administration, the more symptoms severity. In Sener’s study, succinylcholine post-poisoning administration was accompanied by 7-hour apnea (13), whereas it was less (45 minutes) in the present study due to 6-day interval between organophosphate exposure and succinylcholine administration. Acetylcholinesterase level is genetically low in some cases, and there is a report by Williams and colleagues in this area about a patient with cholinesterase deficiency who developed apnea after succinylcholine administration for ECT (17).

Continuous mechanical ventilation until complete restoration of muscle tone is the safest treatment approach for post-succinylcholine-injection apnea (18). Ventilation shall be continued with sufficient sedation (19) and treatment based on oxygenation with 100% oxygen, blood transfusions or FFP or human acetylcholine esterase administration (20).

## Authors' contributions

SM carried out the clinical case report and wrote the manuscript. All authors participated in the acquisition and interpretation of clinical data, read and approved the final manuscript

## Notes

**Declaration of interest**: None.

## References

[1] Rotenberg M, Shefi M, Dany S, Dore I, Tirosh M, Almog S (1995). Differentiation between organophosphate and carbamate poisoning. Clin Chim Acta.

[2] Ebrahimzadeh MA, Shokrzadeh M, Bioukabadi M (2005). [Effect of organophosphorus pesticides on acetyl cholinesterase activity in agricultural workers.]. J Shahrekord Univ Med Sci.

[3] Watson WA, Litovitz TL, Rodgers GC, Klein-Schwartz W, Reid N, Youniss J (2003). 2002 Annual Report of the American Association of Poison Control Centers Toxic Exposure Surveillance System. Am J Emerg Med.

[4] Wu ML, Deng JF, Tsai WJ, Ger J, Wong SS, Li HP (2001). Food poisoning due to methamidophos-contaminated vegetables. J Toxicol Clin Toxicol.

[5] Eddleston M, Phillips MR (2004). Self poisoning with pesticides. BMJ.

[6] Eyer P (2003). The role of oximes in the management of organophosphorus pesticide poisoning. Toxicol Rev.

[7] Khurana D, Prabhakar S (2000). Organophosphorus intoxication. Arch Neurol.

[8] Pope CN, Chakraborti TK (1992). Dose-related inhibition of brain and plasma cholinesterase in neonatal and adult rats following sub lethal organophosphate exposures. Toxicology.

[9] Worek F, Koller M, Thiermann H, Szinicz L (2005). Diagnostic aspects of organophosphate poisoning. Toxicology.

[10] Slotkin TA, Seidler FJ (2007). Comparative developmental neurotoxicity of organophosphate in vivo: Transcriptional response of pathways for brain cell development, cell signaling, citotoxicity and neurotransmitter systems. Brain Res Bull.

[11] Savolainen KM, Hirvonen MR (1992). Second messengers in cholinergic induced convulsions and neuronal injury. Toxicol Lett.

[12] Tuovinen K (2004). Organophosphate induced convulsions and prevention of neuropathological damages. Toxicology.

[13] Sener EB, Ustun E, Kocamanoglu S, Tur A (2002). Prolonged apnea following succinylcholine administration in undiagnosed acute organophosphate poisoning Acta Anaesthesiol Scand.

[14] Barash PG, Cullent BF, Stoelting RK, Lippincott Williams & Wilkins (2001). Clinical anesthesiology.

[15] Lang C, Lukasewitz P, Wulf H, Geldner G (2002). Plasma cholinesterase variations as a result of prolonged neuromuscular blockade. Review and problems encountered in two cases of prolonged neuromuscular blockade after muscle relaxation with succinylcholine as compared to mivacurium. Anaesthesist.

[16] Waghmare A, Kumar CN, Thirthalli J (2010). Suxamethonium induced prolonged apnea in a patient receiving electroconvulsive therapy. Gen Hosp Psychiatry.

[17] Williams J, Rosenquist P, Arias L, McCall WV (2007). Pseudocholinesterase deficiency and electroconvulsive therapy. J ECT.

[18] Lovely MJ, Patterson SK, Beuerlein FJ, Chesney JT (1990). Perioperative blood transfusion may conceal atypical psendocholinesterase. Anesth Analg.

[19] Lang C, Lukasewitz P, Wulf H, Geldner G (2002). Plasma cholinesterase variations as a result of prolonged neuromuscular blockade. Review and problems encountered in two cases of prolonged neuromuscular blockade after muscle relaxation with succinylcholine as compared to mivacurium. Anaesthesist.

[20] Pujic B, Bekvalac M, Kolak R, Ziramov J (1998). Prolonged apnea after administration of succinylcholine. Med Pregl.

